# Mitochondrial transplantation: the advance to therapeutic application and molecular modulation

**DOI:** 10.3389/fcvm.2023.1268814

**Published:** 2023-12-15

**Authors:** James D. McCully, Pedro J. del Nido, Sitaram M. Emani

**Affiliations:** ^1^ Department of Cardiac Surgery, Boston Children’s Hospital, Boston, MA, United States; ^2^ Harvard Medical School, Boston, MA, United States

**Keywords:** mitochondria, mitochondrial transplantation, heart, transcriptomics, proteomics

## Abstract

Mitochondrial transplantation provides a novel methodology for rescue of cell viability and cell function following ischemia-reperfusion injury and applications for other pathologies are expanding. In this review we present our methods and acquired data and evidence accumulated to support the use of mitochondrial transplantation.

## 1. Introduction

The heart is an obligate aerobic organ and is dependent upon oxygen delivery to the mitochondria to ensure function. Mitochondria represent approximately 30% of cardiomyocyte cell and extract >75% of coronary arterial oxygen just to meet homeostatic requirements (1). Increased functional demands are therefore dependent on increased oxygen delivery either through increased coronary blood flow and/or oxygen extraction.

Interruption or limitation in coronary blood flow limits oxygen delivery to the myocardium such that it is no longer sufficient to meet metabolic demands and results in myocardial ischemia. It is generally accepted that the limitation or cessation of coronary blood flow to the myocardium is the initial step in the myriad of processes leading to myocardial ischemia injury and these processes primarily effect the mitochondria (2–4).

With the onset of myocardial ischemia, alterations occur in mitochondrial structure and function that negatively impact mitochondrial function (5–8). The mitochondria become swollen and there is cristae disruption and mitochondrial calcium accumulation and the electron transport chain complexes I-V show decreased activity leading to decreased high energy phosphate synthesis that is needed to maintain cellular function. Mitochondrial DNA is also damaged leading to decreased mitochondrial transcriptomics, proteomics and metabolomics and the intrinsic apoptotic pathway is activated, leading to loss of cellular viability (6, 8–11). Accompanying these changes are alterations in transcriptomic, proteomic and metabolomic pathway regulation that are directly associated with mitochondrial and contractile function (12–15). All these events occur during ischemia and despite the restoration of coronary blood flow and oxygen delivery to the myocardium, they persist during reperfusion to significantly compromise myocardial cellular viability and function (8–10).

Interventions to limit mitochondrial dysfunction during ischemia and reperfusion have mainly been directed to mechanisms or pathways up- or down-stream of the mitochondrion. While somewhat efficacious, these interventions have provided only minimal clinical utility for the amelioration of the effects of ischemia-reperfusion injury. As an alternative we have proposed organelle transplant, mitochondrial transplantation, to directly address mitochondrial dysfunction. Mitochondrial transplantation is premised on the observed alterations in mitochondrial function that manifest during ischemia and persist through reperfusion. We hypothesized that the replacement or augmentation of damaged mitochondria through the transplantation of viable, respiration competent mitochondria, isolated from non-ischemic tissue, and then delivered to the ischemic organ would enhance post-ischemic myocardial functional recovery and myocellular viability (14).

Mitochondrial transplantation provides a novel methodology for the rescue of cell viability and cell function following ischemia-reperfusion injury and applications for other pathologies are expanding. A systematic review of animal and human studies supports the beneficial effects of mitochondrial transplantation for the amelioration of ischemia- reperfusion injury (16). In this review we present our methods and acquired data and the accumulated evidence to support the use of mitochondrial transplantation.

## 2. Mitochondrial uptake and functional integration

The earliest example of naked mitochondrial uptake into cells was reported by Clark and Shay (17). The authors used simple coincubation of isolated mitochondria from antibiotic resistant cells with antibiotic sensitivity to show that antibiotic resistance could be transferred. The authors showed that the antibiotic resistant mitochondria were taken up by the antibiotic sensitive cells by endocytosis and that the transferred mitochondria were functional and conferred antibiotic resistance. These early studies, termed mitochondrial transformation by the authors, were mostly observational and were posited as a novel means for studying mitochondrial genetics in mammalian cells and provided early seminal evidence for the uptake and functional integration of exogenous mitochondria.

The uptake of mitochondria into cells has been demonstrated by numerous authors using a variety of methods (18–23). Katrangi et al. (18) showed that co-incubation of isolated mitochondria from human mesenchymal stem cells (hMSC) with non-respiration functional A5490 *p*^0^ cells having fully depleted mtDNA, rescued cell function and restored cellular respiration. Uptake was confirmed by fluorescent labelling and PCR analysis.

Kitani et al. (19) used DsRed2 mitochondria isolated from human uterine EMCs-DsRed2 cells to demonstrate uptake and functional integration of mitochondria into recipient H9c2 cells, stably expressing green fluorescent protein (GFP). The authors used co-incubation and showed the engulfment of exogenous mitochondria. The exogenous mitochondria were evident in the perinuclear space inside the recipient cells within 1–2 h. The transferred mitochondria were able to rescue the mitochondrial respiratory function and improved the cellular viability in mitochondrial DNA-depleted cells and these effects lasted six days.

Pacak et al. (20) also showed that co-incubation of naked mitochondria with non-functional HeLa *p*^o^ cells lacking mitochondrial DNA, resulted in the rapid uptake of the exogenous mitochondria. Uptake was visualized using mitochondria labelled with pHrodo a label that specifically detects phagocytosis and endocytosis with a pH-sensitive fluorogenic dye that is non-fluorescent at neutral pH and bright red upon acidification inside the cytosol. The internalized mitochondria rescued mitochondrial oxygen uptake and ATP synthesis and replaced mitochondrial DNA. These effects were present for 53 cell divisions over 23 days.

Cowan et al. (24) used three-dimensional super-resolution structured illumination microscopy (3-D SR-SIM) and transmission electron microscopy (TEM) to reveal the intracellular position of endocytosed mitochondria in human induced pluripotent stem cell-derived cardiomyocytes and human cardiac fibroblasts. These studies used a human cardiac fibroblast cell line as the source of mitochondria for transplantation and a human iPS cardiomyocyte cell line as the recipient cell line to demonstrate the uptake and functional integration of mitochondria into human cells.

Cowan et al. (24) used distinct fluorescent labeling of human induced pluripotent stem cell-derived cardiomyocytes and human cardiac fibroblasts using baculovirus-mediated transfer of mammalian fusion genes containing fluorescent probes (green; GFP or red; RFP) fused to the leader sequence of E1 alpha pyruvate dehydrogenase. Pyruvate dehydrogenase is a matrix associated protein and fusion labeling provides a reliable methodology for imaging and detection. The authors labeled mitochondria in human induced pluripotent stem cell-derived cardiomyocytes with RFP while mitochondria in human cardiac fibroblasts were labeled with GFP.

Cowan et al. (24) co-incubated the GFP labeled mitochondria, isolated from human cardiac fibroblasts with human induced pluripotent stem cell-derived cardiomyocytes containing RFP labeled mitochondria. These experiments were replicated by coincubation of human induced pluripotent stem cell-derived cardiomyocytes with mitochondria isolated from human cardiac fibroblasts labelled with gold nanoparticles with subsequent TEM analysis.

Cowan et al. (24) showed that mitochondrial uptake into cells is rapid and can be seen at 2.5 min post-delivery, the shortest time frame allowable for experimental determination. The authors showed that the transplanted mitochondria were detected adjacent to the apical cell surface, undergoing endocytosis and then being taken up and released from early and late endosomes and then fusing with intrinsic mitochondria within the cell. The transplanted mitochondria were of the proper size and shape and contained the mitochondrial fusion proteins MFN1, MFN2 and OPA1. A small amount of DRP1 was detected but was not phosphorylated, suggesting fission did not occur. Greater than 80% of the transplanted mitochondria could be detected in association with early endosomes and late endosomes and then released into the cell to fuse with the endogenous mitochondria. Greater than 70% of the endocytosed mitochondria co-localized and fused with endogenous mitochondria.

Kesner et al. (21) have also shown mitochondrial uptake into cells. In these studies mitochondria labelled with DsRed were co-incubated with HepG2 cells where they fused with the intrinsic mitochondria. The authors reported that internalization of exogenous mitochondria can occur in as little as 10 min and showed that uptake of the exogenous mitochondria lasted for at least 6 days. Further experiments using patient cells showed the transplanted mitochondria increased cell viability and mitochondrial activity.

These studies have been recently confirmed by Rossi et al. (25) who have also shown that co-incubation of isolated mitochondrial with recipient cells results in the internalization of isolated mitochondria. The authors demonstrated that mitochondria isolated from renal proximal tubular cells were biologically active and capable of ATP production and that the isolated mitochondria could be actively internalized by renal proximal tubular cells in a dose dependent manner. The transplanted mitochondria increased proliferative capacity and ATP production and proliferation and significantly decreased cytotoxicity in an *in vitro* ischemia-reperfusion injury model.

Ali-Pour et al. (23) were also able to demonstrate mitochondria uptake into cardiomyocytes; however, in contrast to reports by others, increased bioenergetics lasted only 2 days (13, 19–21, 26, 27).

In our early experiments we used fluorescent mitochondrial specific labels such as MitoTracker CMXros and pHrodo to demonstrate mitochondrial uptake into cells (14, 20). The use of these fluorescent labels is informative but is not definitive as dissociation and re-association events have been postulated to occur.

To unequivocally demonstrate mitochondrial uptake, we have used human mitochondria for transplantation into animal models (13, 15, 26–30). The use of xenogeneic human mitochondrial transplantation in a rat, murine or swine model allows for the differentiation between native mitochondria and transplanted mitochondria based on immune reactivity to a monoclonal anti-human mitochondria antibody (24, 27). The use of human mitochondria in the rabbit and swine heart, kidney, lung and skeletal muscle has allowed us to track the fate of transplanted mitochondria across time. We use immunohistochemical selectivity to the human mitochondrial antibody as our primary marker with secondary markers of size and shape and function to confirm mitochondrial uptake. The transplanted human mitochondria in the rabbit and swine heart, kidney, lung and skeletal muscle induced no immune response as determined by ELISA and multiplex analysis and appear to maintain viability. Increased ATP content was detected at both 2 h and at 28 days after transplantation in the areas of mitochondrial transplantation (13, 15). No DAMPs (damage-associated molecular patterns) response or apoptosis or necrosis is evident in the areas receiving xenogeneic mitochondrial transplantation (13, 15, 26, 31).

It must be clearly noted that we do not recommend xenogeneic mitochondrial transplantation as the mtDNA differs and sufficient mitochondrial sources are available such that arguments for xenogeneic mitochondrial transplantation are moot (32).

### 2.1. Stability of functional integration

Our *in vivo* studies have shown that mitochondrial uptake is stable and can be visualized for at least 28 days post-transplantation in *in vivo* transplantation (13, 15, 26). The transplanted mitochondria are evident in both myocardial and non-myocardial cells at 2, 4, 8 and 24 h and at 28 days post-delivery. The exogenous mitochondria enhance cardiac function (increased left ventricular pressure, systolic shortening, decreased end diastolic pressure), enhance tissue viability (decreased tissue caspase 3 activity and necrosis) and enhance total tissue energy content (increased total tissue ATP content) following transplantation (13, 15, 26). These effects are evident at 2 h reperfusion and at 28 days recovery. Significantly our results show that there is no increase in peri-infarct size and that functional improvements in myocardial contraction remain intact throughout the recovery time of 28 days, verifying the enduring effects of mitochondrial transplantation on cellular viability and function.

### 2.2. Mechanism of uptake

The mechanisms for mitochondrial transplantation are distinct from mitochondrial transfer (20). Mitochondrial transfer involves the horizontal transfer of mitochondria from one cell to another. The transfer of mitochondria has been shown to occur through tunnelling nano tubes (TNT) which can occur either by uni- or bi-directional transfer (33). Spees et al. (34) demonstrated that co-culture of A549 cells with non-functional A549 *p*^o^ cells lacking mitochondrial DNA resulted in some of the non-functional cells acquiring functional mitochondria. This rescue was shown to occur through active mitochondrial transfer along cytoplasmic projections that made contact between donor and target cells. The authors were not able to establish whether mitochondria were transferred to the target cells directly through structures such as tunneling nanotubes or through uptake of vesicles containing mitochondria that budded off from the donor cells. Interestingly the authors showed that there was no passive transfer of mitochondria. Naked mitochondria isolated by differential centrifugation did not provide for rescue of A549 *p*^o^ cells.

Liu et al. (33) and Han et al. (35) were able to detect TNT-like structures allowing for intracellular transfer of mitochondria. Further studies by Berridge and Tan (36) and Tan et al. (37) confirmed the acquisition of mtDNA from host cells. Hayakawa et al. (22) have also shown that mtDNA and intact mitochondria can be transferred from other cells. These authors showed that extracellular mitochondria from astrocytes rescued neuronal viability and function.

## 3. Mitochondrial transplantation

### 3.1. Tissue source

The need for viable respiration competent mitochondria is essential for mitochondrial uptake and functional integration. In our initial publication on mitochondrial transplantation for cardioprotection we showed that the use of frozen mitochondria with reduced mitochondrial oxygen consumption and membrane potential did not provide for ischemia- reperfusion protection (14). We also showed that the use of mitochondrial proteins, mitochondrial RNA and DNA or ATP did not provide for cardioprotection (14). These findings have been confirmed by Hayashida et al. (38) who have shown that frozen-thawed mitochondria have reduced membrane potential and were not efficacious for resuscitation following cardiac arrest in rats. This agrees with Kesner et al. (21) who have shown that disruption of the mitochondrial membrane decreases uptake of mitochondria into recipient cells. The importance of having intact respiration competent mitochondria has also been demonstrated by Cloer et al. (39) who have confirmed these earlier findings in a human DCD lung transplantation model where they showed that only intact mitochondria and not organelle secretions provided for therapeutic activity.

To allow for mitochondrial transplantation an appropriate tissue source must be available. Autologous tissue obtained from a non-ischemic site from the patient's own body offers the most clinically relevant source. In our studies, the source of tissue for mitochondrial isolation varies depending upon the incision site required for surgical access. This allows for therapeutic application without the need for secondary surgical intervention. In our procedures where a mini-thoracotomy or a sternotomy is performed, tissue from the pectoralis major or the rectus abdominus is obtained. When a carotid cut down is performed, tissue from the sternocleidomastoid can be obtained or when a femoral cut down is performed, tissue from the vastus medialis can be obtained. Other sources of tissue would depend on the incision site. The use of liver tissue may be appropriate when a laparotomy is performed.

A variety of cell lines have also been used as the source material for mitochondrial isolation. Pacak et al. (20) used mitochondria isolated from HeLa cells, Ali Pour et al. (23) used L6 skeletal cells, while Chang et al. (40) and Gollihue et al. (41) each used PC12 cells and Caicedo et al. (42) have used mitochondria isolated from mesenchymal stem cells. The potential for heterologous sources of tissue is great and could allow for readily available application in clinical settings and could allow for therapeutic treatment of mitochondrial associated mitochondrial myopathies.

We have used atrial appendage tissue, skeletal muscle, liver and cell culture as source material for mitochondrial isolation. We have found no advantage using organ specific or high or low glycolytic capacity mitochondria for ischemia-reperfusion protection (13–15, 20, 24, 26, 27).

Once exposed the tissue is dissected from the skeletal muscle using a number 6 biopsy punch. Usually two small pieces of tissue (>0.1 g) are harvested and stored in cold (4°C) phosphate buffered saline (clinical grade) and used for mitochondrial isolation. There are many methods for the isolation of mitochondria. The earliest published accounts of mitochondrial isolation date to the 1940s and these methods have been expanded upon and modified (43–45). Most mitochondria isolation protocols use tissue homogenization followed by differential centrifugation (46–48). Purification by Percoll gradient or sucrose step gradient centrifugation is often incorporated to purify the isolated mitochondria; however, the added centrifugation and washing time greatly extends the isolation process (49, 50).

### 3.2. Mitochondrial isolation

**Figure 1:**
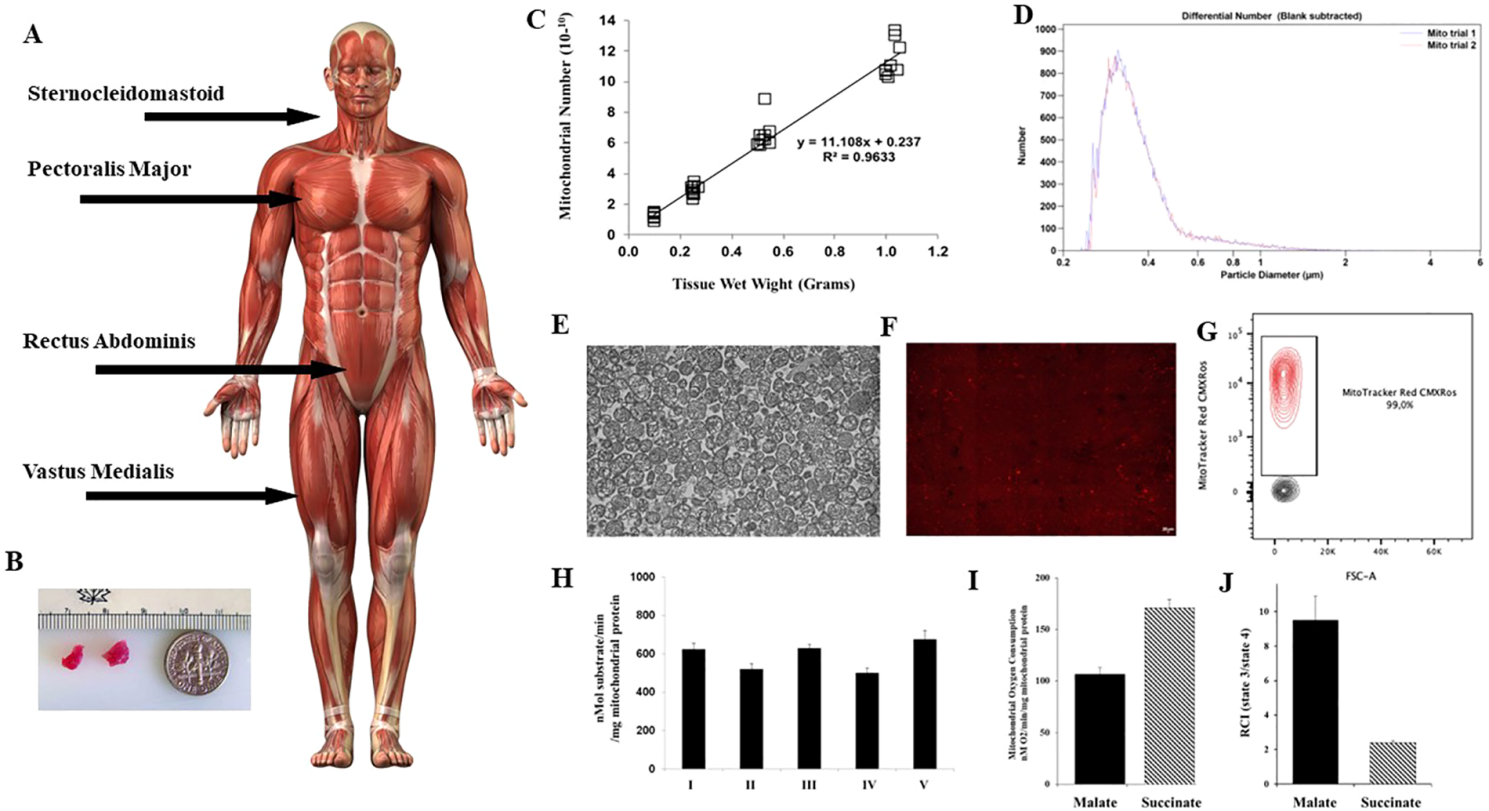
Mitochondria are isolated from tissue obtained at the site of the surgical incision. (**A**) Skeletal muscle tissue for mitochondrial isolation is shown at incision sites commonly used in cardiac surgery for sternotomy and mini thoracotomy. (**B**) Tissue is obtained using a #6 biopsy punch. Only two small pieces of viable tissue are needed. (**C**) From these two small pieces of tissue weighing approximately 0.1 gram 1 × 10 10 mitochondria can be obtained using the isolation methodology as described by (53, 54), and described at Available at: https://sites.google.com/mccullylab.org/mccullylab. (**D**) The isolated mitochondria are of consistent size as determined by particle counter. (**E**) The purity of the isolated mitochondria can be seen by transmission electron microscopy. (**E**) The mitochondria maintain membrane potential as determined by MitoTracker Red CmxRos staining. (**F**) Flow cytometry analysis of isolated mitochondria stained with Mitotracker Red CMXRos shows 98.6% of isolated mitochondria maintain membrane potential. (**H**) Complex I-V activity is maintained and (**I**) Mitochondrial oxygen consumption and (**J**) Respiratory Control Index (state 3/state 4) for malate induced (complex I) and succinate induced (complex II) is preserved.

In our initial studies we isolated mitochondria using differential centrifugation (47, 48). The isolation of mitochondria was completed in approximately 90 min and required a starting tissue of approximately 5 grams (14). This amount of tissue is not available clinically and the viability of the isolated mitochondria using these methods is variable (51), for a more in-depth review of mitochondrial isolation techniques the reader is directed to (46–48, 52, 53). In addition, the time required for the isolation of mitochondria using this methodology would require extension of the surgical time and could result in complications that would be injurious to the patient (Figure 1).

To meet clinical demands, we have developed a novel methodology that allows for the rapid isolation and purification of mitochondria using differential filtration (53, 54). This methodology uses mechanical homogenization to minimize operator variability of homogenization. The tissue is obtained from the site of incision and is homogenized in a volume of 5 ml of sterile isolation buffer consisting of 300 mmol/L sucrose, 10 mmol/L HEPES-KOH [4-(2-hydroxyethyl)-1-piperazineethanesulfonic acid—potassium hydroxide], 1 mmol/L EGTA-KOH (Ethylenediaminetetraacetic acid—potassium hydroxide), pH 7.4, and then treated to 10 min of Subtilisin A enzymatic digestion, on ice. The digested tissue is then filtered through a series of filters by gravity filtration, and the mitochondria are subsequently precipitated by centrifugation at 9.5 × G for 5 min at 4°C (53, 54), https://sites.google.com/mccullylab.org/mccullylab). The time required for mitochondrial isolation using this procedure is 20–30 min and does not delay the surgical procedure.

The usual mitochondrial number obtained from the tissue samples (>0.1 g) using this methodology is 0.5–1.0 × 10^10^ mitochondria. The isolated mitochondria are of the correct size and shape, as assessed by particle size counter and by transmission electron microscopy and have normal cristae and membranes and show no damage or injury (13, 24, 27, 28). The isolated mitochondria maintain membrane potential and oxygen consumption as determined by MitoTracker Red CMXRos staining and FACs analysis and mitochondrial complex I-V activity is maintained.

The isolated mitochondria have no detectable cytosolic, nuclear, or microsomal components that would include fragments from endoplasmic reticulum, endosomes, golgi apparatus, nucleus, and cytosol. We have performed enzymatic analysis of mitochondria isolates for detection of cytosolic and cytoplasmic contaminant markers glyceraldehyde 3-phosphate dehydrogenase and lactate dehydrogenase and the microsome contaminate markers 5´nucleotidase and glucose-6-phosphatase, and have found no detectable contamination by any of these contaminants. Western blot analysis also showed no detectable presence of any contaminant markers in the isolated mitochondrial preparations (13, 24, 27, 28).

In all our studies, we have used total mitochondria for mitochondrial transplantation. The bioenergetic function of this population includes that of sub-sarcolemmal and intra-fibrillar mitochondria (55–57). We have found that total mitochondria offer the same level of cardioprotection as that provided by purified intrafibrillar or subsarcolemmal mitochondria sub-populations (14). The ease of obtaining total mitochondria and the reduced time for isolation make this choice clinically relevant.

### 3.3. Mitochondrial buffer for delivery

Following isolation, the mitochondria are suspended in buffer. In our early studies we suspended the isolated mitochondria in respiration buffer. This buffer contained 0.25 M sucrose, 0.002 M KH_2_PO_4_ (potassium dihydrogen phosphate), 0.1 M MgCl_2_ (magnesium chloride), 0.2 M HEPES-KOH (pH 7.6), 0.0005 M EDTA-KOH (pH 8.0), 0.005 M glutamate, 0.005 M malate, and 0.001 M ADP (adenosine diphosphate) (14). Respiration buffer needs to be made fresh as the glutamate, malate and ADP can be degraded. Following several trials, we found that we could suspend the mitochondria safely in isolation buffer (300 mmol/L sucrose, 10 mmol/L HEPES-KOH, 1 mmol/L EGTA-KOH, pH 7.4) and that the isolated mitochondria maintained viability and function (15, 28, 58–60). The use of isolation buffer allows for ease of use in clinical applications as the multiple steps needed to make “fresh” respiration buffer are not required.

Modifications of this buffer have been used successfully by others. Hayashida et al. (38) have used buffer containing 250 mM sucrose, 2 mM KH_2_PO_4_, 10 mM MgCl_2_, 20 mM K-HEPES-KOH (pH 7.2), 0.5 mM EGTA-KOH (pH 8.0). Huang et al. (61) have used 70 mM sucrose, 220 mM mannitol, 10 mM KH_2_PO_4_, 5 mM MgCl_2_, 2 mM HEPES, 1.0 mM EGTA and pH 7.2, for their studies in the rat. Gollihue et al. (41) suspend their isolated mitochondria in 215 mM mannitol, 75 mM sucrose, 0.1% bovine serum albumin, 20 mM HEPES, pH adjusted to 7.2 with KOH.

### 3.4. Stability after isolation

**Figure 2:**
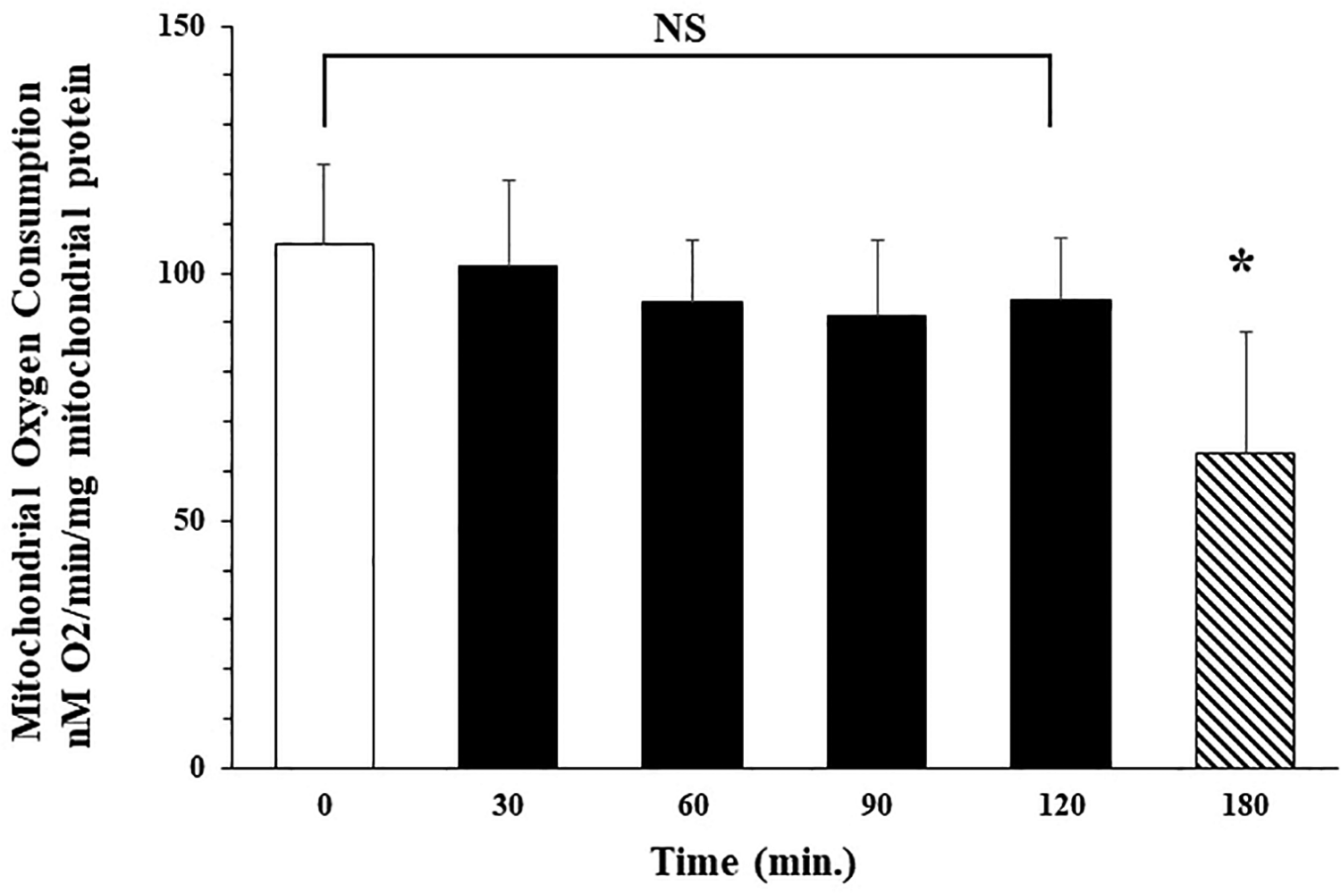
Isolated mitochondria are stable at 4°C for 120 min No significant difference (NS) was observed in mitochondrial oxygen consumption at 0, 30, 60, 90 or 120 min after isolation. Mitochondrial oxygen consumption significantly decreases at 180 min * = *p* < 0.05 vs. time 0.

Our studies demonstrate that following isolation, oxygen consumption rate in isolated mitochondria is stable for at least 2 h with storage on ice. Mitochondrial state 3 oxygen consumption for malate (complex I) immediately following isolation was 106.6 ± 16.0 (nM O_2_/min/mg mitochondrial protein). No significant difference in oxygen consumption rate was observed at 30, 60, 90, or 120 min after isolation with storage on ice. Oxygen consumption rate was 101.6 ± .17.3; 94.3 ± 12.3; 91.3 ± 15.6; and 94.6 ± 12.6 nM O_2_/min/mg mitochondrial protein respectively. Oxygen consumption rate at 3 h storage was significantly decreased to 63.6 ± 24.6 nM O_2_/min/mg mitochondrial protein. These results demonstrated that autologous mitochondria isolated using our methodology, in less than 30 min, were viable and suitable for clinical use for up to 2 h following isolation when stored on ice (Figure 2, unpublished data McCully et al.).

At present, only autologous tissue and cell culture have been used as sources for mitochondria isolation for use in mitochondrial transplantation. Cell culture as a source for mitochondrial isolation could provide a ready and available source of mitochondria that could be used for immediate use in settings such as acute heart attack. The best scenario would be to have an “of-the-shelf” product that would allow for immediate usage, however, the methodology for such a product remains to be developed.

### 3.5. Delivery methods

A variety of delivery methods for mitochondrial transplantation have been developed and these range from simple naked co-incubation to sophisticated mechanical delivery. Chang et al. (40) have proposed the use of a cell-penetrating peptide (Pep-1) to aid in the uptake and internalization of mitochondria. The authors used Pep-1 conjugated mitochondria in a rat model of Parkinson's disease and have reported improvement of rotational and locomotor behaviors in this model.

Liu et al. (62) have developed a method where isolated mitochondria are linked to a carrier that allows the systemically injected mitochondria to be directed to the liver. The authors used asialoglycoprotein linked to listeriolysin O to form complexes with freshly isolated liver mitochondria. This complex forms a mitochondrial-carrier protein capable of being recognized, bound, and internalized by asialoglycoprotein receptors in the liver. Listeriolysin O was added to facilitate the release of internalized mitochondria from endosomes. Using this complex the authors reported targeted delivery of mitochondria by intravenous delivery in the rat with 27% of the injected mitochondria being found in the liver.

Kim et al. (63) have shown in cell cultures that isolated mitochondria can be transferred into recipient cells by simple low speed [1,500  ×  g (gravity) for 5 min at 4°C.] centrifugation. The transferred mitochondria maintained bioenergetic function and increased intracellular ATP content and metabolic activity, and also delivered mtDNA to the recipient cells.

MitoCeption, proposed by Caicedo et al. (42) also uses centrifugation but adds thermic shock to enhance mitochondria uptake in cells. The authors added isolated mitochondria to cells grown on a cell plate surface. The culture plates were then centrifuged at 1,500 × g for 15 min at 4°C. They were then placed in a 37°C cell incubator for two hours, prior to a second centrifugation. The authors showed that MitoCeption increased mitochondrial DNA concentrations, oxygen consumption rate and ATP production.

Mechanical approaches have also been proposed to facilitate mitochondrial delivery. Wu et al. (64) have proposed the use of a photo-thermal nanoblade which induces a transient membrane opening to allow for mitochondrial uptake into cells. These transient and localized openings are thought to allow for specific uptake of mitochondria. In another modification for mitochondrial uptake Wu et al. (65) have proposed the use of a stamp-type multi-needle injector. The device has been used for the delivery of mitochondria to restore aging-related hair loss.

Macheiner et al. (66) have developed Magnetomito Transfer which uses a mitochondrial specific antibody to TOM-22 linked to an iron bead that binds to the outer mitochondrial membrane to stimulate mitochondrial uptake under a magnetic field. The methodology was developed to allow for transfer of healthy allogenic mitochondria to a patient's own stem cells prior to autologous stem cell transplantation. The authors report a trend for higher density of mitochondria in magnetomito-transferred cells.

Recently, Yang et al. (67) have suggested the use of gelatin nanospheres to enhance mitochondrial uptake into cells. In their studies isolated mitochondria were first modified by electrostatic attachment of gelatin nanospheres to the outside of the mitochondria. The nano-sphere-mitochondria were then co-incubated with H9c2 cardiomyoblasts where functional uptake was confirmed by ATP synthesis.

Another approach to delivery of genes or pharmaceuticals to the mitochondria is Mito-porter developed by Yasuzaki et al. (68). This methodology does not use mitochondria but rather uses a liposome-based carrier that permits macromolecular cargos to enter mitochondria via membrane fusion. The authors have used mito-porter in a series of studies to demonstrate that mtDNA and other products can be delivered effectively to the mitochondria (69).

#### 3.5.1. Co-incubation

For cell culture studies, most authors resuspend the isolated mitochondria in media. In our studies the cell culture media Dulbecco's Modified Eagle's Medium contains 1.8 mM calcium, We and others (13, 20, 21, 70) have found no stability problems of the isolated mitochondria in these physiological calcium concentrations.

### 3.6. Stability in Serum and blood

Shi et al. (71) investigated isolated mitochondrial stability in rat serum. The authors added isolated mitochondria to mouse blood serum and incubation at the mixture at 37°C for 0, 15, 30, 60, 120 min, respectively. The authors then measured mitochondrial viability using MitoTracker Red CMXRos and mitochondrial membrane potential at each time point. The authors reported that there was no difference in mitochondrial viability or mitochondria membrane potential (ΔΨm) observed at the different time points (0 min, 15 min, 30, 60 and 120 min) suggesting that the mitochondria remained stable in serum for at least 120 min.

Intact mitochondria have been detected in plasma in healthy patients and in patients having pathological conditions. Proof for the existence of cell-free respiratory competent mitochondria in blood was first reported by Amir Dasche et al. (72). The authors showed mitochondria from a normal cell line (CCD-18Co) and from human colon cancer cell lines (DLD-1/SW620) were able to secrete their mitochondria. The authors went on to show the presence of circulating cell-free mitochondria in healthy individuals and in cancer patients. The cell free circulating mitochondria were structurally intact and not fragmented and had preserved mtDNA integrity and were not surrounded by bi- or multi-layer phospholipid membrane, supporting the observation that they were free circulating mitochondria and not enclosed in extracellular vesicles such as exosomes or microvesicles or autophagosomes. The cell free circulating mitochondria were also respiration competent.

These findings have been replicated by Stephens et al. (73) who showed that intact, cell free mitochondria are present in blood and these mitochondria maintained transmembrane potential and were able to reenter cells.

Amir Dasche et al. (72) and Stephens et al. (73) estimated the presence of cell-free and respiratory competent mitochondria in human blood to be between 200,000 to 3.7 million per mL and 822,000 to 2.3 million per mL plasma in humans, respectively. The mechanisms through which mitochondria are able to survive in serum and their relevance in non-pathological human organ maintenance are unknown.

### 3.7. Direct injection

Direct injection into the tissue is the simplest methodology for clinical delivery of mitochondria for transplantation. For direct injection, the isolated mitochondria are suspended in buffer and are delivered to the organ using a 1 ml tuberculin syringe with a 28 or 32 gage needle. The mitochondria can be delivered directly to affected areas in the organ. In the heart this may be areas of hypo-kinesis or a-kinesis as determined by epicardial echocardiography. The mitochondria are delivered in 50–100 ul injections. This volume is rapidly taken up by the neonatal, pediatric and adult myocardium and no flushback occurs and there is no need for purse string sutures. Similar uptake is observed in skeletal muscle. The angle of injection is not limited to oblique delivery as in stem cell injection (13–15, 26, 27, 29, 30, 74, 75).

The isolated mitochondrial dosage amounts in the heart were determined to be 2 × 10^5^ to 2 × 10^6^ mitochondria per gram tissue wet weight (13, 14, 28). Mitochondrial concentrations >2 × 10^8^ were not consistently fully suspended in vehicle buffer.

Direct injection of mitochondria has no effect on arrhythmogenicity (13). Our studies in the *in vivo* heart have demonstrated that there is no proarrhythmia as determined by serial ECG, no change in QRS duration and no change in corrected QT interval. Serial electrocardiography and echocardiography also demonstrated that there was no evidence of wall motion disturbances, no evidence of left ventricle hypertrophy, valve dysfunction, fibrosis or pericardial effusion at 4 weeks following transplantation of mitochondria.

To ensure no proarrhythmia was associated with mitochondrial transplantation we also performed optical mapping studies using isolated mitochondria at a concentration of 8.4 × 10^7^/gram tissue wet weight as compared to 2 × 10^5^ to 2 × 10^6^/gram heart wet weight. This concentration of mitochondria was used in order to detect any possible arrhythmogenic response (13). Our results demonstrated that there was no change in isopotential mapping associated with mitochondrial transplantation and confirming that mitochondrial transplantation is not proarrhythmic.

### 3.8. Intra-arterial delivery

Intra-arterial delivery of mitochondria simplifies mitochondrial delivery to tissue and allows for widespread distribution of mitochondria within the tissue. Intra-artery delivery to the heart is achieved via carotid cutdown (28). For the lung we deliver via the pulmonary artery (76) while for the kidney we perform a femoral artery cutdown (29).

The artery is exposed and accessed, and an arterial sheath is positioned in the artery and an angiography catheter is introduced through the arterial sheath, and floated to the delivery site, the coronary ostium in the case of the heart or the infrarenal arteries in the case of the kidney. This procedure is performed under fluoroscopy (28, 29, 58, 77). A 4 s or 5s catheter can be used. The isolated mitochondria are suspended in 5 ml of buffer and injected as a bolus and then chased with 5 ml of buffer.

To ensure that intra-coronary delivery was safe and did not affect coronary patency we have performed *in vivo* studies in the pig heart (28). The pig heart provides a standardized model for the human heart having similar vascular architecture, capillary diameter and morphology as that in the human heart (78, 79). In these studies, intra-coronary delivery was investigated using mitochondria concentration of 1 × 10^3^ to 1 × 10^11^ in the presence of increased myocardial demand, coronary vasoconstriction or tachycardia with increased afterload. Our results demonstrated that intracoronary injection of mitochondria at concentrations of 1 × 10^3^ to 1 × 10^11^ has no adverse effects on coronary patency, cardiac rhythm, or function. We also showed that mitochondria can be safely injected into severely constricted coronaries as well as under significant hemodynamical stress of tachycardia and hypertension, all of which often accompany various pathological conditions of the heart. As with direct injection of mitochondria there is no proarrhythmia associated with intra-arterial delivery of mitochondria.

### 3.9. Aerosol delivery

To increase applicability and allow for early, non-surgical intervention and post-surgical intervention we have extended our research to show that autologous mitochondria can be delivered by nebulizer to the lungs. In a study of acute lung injury, we compared aerosol delivery of naked mitochondria via the trachea and intra-vascular delivery, via the pulmonary artery (76). Vascular delivery of mitochondria to the left lung was achieved by injection of mitochondria in buffer directly into the left pulmonary artery. Aerosol delivery of mitochondria was achieved by nebulization using the FlexiVent nebulizer system (FlexiVent FX2, SCIREQ, Montreal, Quebec, Canada). Our results demonstrated that delivery of mitochondria by pulmonary artery infusion and by nebulization was efficacious.

### 3.10. Nasal delivery

In the brain, delivery of exogenous mitochondria to the ventricles has been shown to be effective, but clinically, the impermeability of the blood-brain barrier poses a major restriction for use. Orreogo et al. (80) have reported that the delivery of mitochondria to the brain can be achieved using osmotic disruption of the blood-brain barrier followed by carotid artery infusion of the mitochondria. The authors report that in the absence of osmotic disruption, few mitochondria were detected in the brain but following osmotic disruption, transplanted mitochondria were detected in all regions of the cortex and across the parenchyma.

Alexander et al. (81), and Alexander et al. (82), have shown that nasal administration of mitochondria isolated from human mesenchymal stem cells restored executive functioning, working and spatial memory in mice with cisplatin-induced cognitive deficits. The authors showed human mitochondria gained rapid entry into the brain of mice. The transplanted mitochondria were seen entering the brain from the pia mater v and glial limitans and were present at 30 min and 18 h after administration and repaired cisplatin-induced loss of white matter integrity and synaptic damage. The transplanted mitochondria also induced transcriptomic alterations in the hippocampus, up-regulating cognitive related restorative pathways. These findings agree with Chiu et al. (83) and Galeano et al. (84) and Danielyan et al. (85) who have each shown uptake of nasal delivered mesenchymal stem cells in the brain.

### 3.11. Delivery to the spinal cord

Delivery of mitochondria to the spinal cord has been shown to be troublesome. Golihue et al. (41) studied mitochondrial transplantation in a rat model of spinal cord injury. The authors showed that exogenous mitochondrial were taken up by the injured spinal cord and were evident at 24- and 48 h, and 7 days post-injection and increased bio-energetics. The mitochondria co-localized with multiple resident cell types, although they were absent in neurons. Unfortunately, mitochondrial transplantation did not yield long-term functional neuroprotection as assessed by overall tissue sparing or recovery of motor and sensory functions. This group have since gone on to promote new techniques for mitochondrial transplantation for spinal cord injury (86).

## 4. Uptake occurs in healthy cells and in ischemic cells

The uptake of mitochondria has been shown in many different cell types and pathologies. In our studies we have found that mitochondria are taken up by all cell types. To visualize mitochondrial uptake and distribution subsequent to direct injection or infusion through the coronary vasculature, we have labelled mitochondria with ^18^F-Rhodamine-6G (^18^F-R6G) or ^18^F-R6G and iron oxide nanoparticles (27–30, 87). The distribution and uptake of labelled mitochondria was determined by positron emission tomography (PET), microcomputed tomography (uCT), and magnetic resonance imaging (MRI) with subsequent microscopic analyses of stained tissue sections to confirm the uptake and distribution of transplanted mitochondria (27). In these studies, we showed that direct injection of mitochondria resulted in discrete localized uptake of the labelled mitochondria while vascular delivery of mitochondria through the coronary arteries resulted in their rapid integration and widespread distribution throughout the heart. Both modes of delivery provided cardioprotection from ischemia-reperfusion injury by significantly preserving systolic shortening and cell viability.

Notably we showed that mitochondria are taken up in myocardial areas that were not subjected to regional ischemia and reperfusion. The uptake in non-ischemic areas was less than that observed in the regional ischemic area in the heart, and we speculate that this increased organelle uptake was due to myocardial cell swelling and loss of cellular integrity.

## 5. Dosage

The dosage of mitochondria varies in different studies. Both mitochondrial protein and absolute mitochondrial number have been used to standardize dosage (13, 19, 21, 23, 41). In all our studies we have used particle counting to estimate the dose of mitochondria used. Our studies have shown that mitochondria concentrations of 2 × 10^5^ to 2 × 10^6^ mitochondria per gram wet weight heart tissue is efficacious (13, 26, 28, 58, 77). This represents approximately 1 × 10^9^ mitochondria in the adult 400 g heart. Concentrations less than 2 × 10^5^ to 2 × 10^6^ mitochondria per gram wet weight heart tissue were associated with decreased cardioprotective efficacy while concentrations greater than 2 × 106 per gram wet weight heart tissue failed to increase efficacy (28). We have found that this dosage concentration is also applicable to the kidney (29) and the lung (76); however, for skeletal muscle increased mitochondrial concentrations were required (30).

Our studies have shown that only a small number of mitochondria are needed to alter organ function. These studies and those of others suggest that the number of mitochondria required for cardioprotection is not a function of the absolute number of mitochondria residing within the cell (14, 28, 88, 89). This would agree with Shoffner et al. (90) who have demonstrated that only 2%–6% of mtDNA needs be wild type to alter oxygen uptake and the devastating effects of the mitochondrial myopathy MERRF (Myoclonic epilepsy and ragged-red fiber disease) and with Chomyn et al. (91) who have reported that levels of only 6% wild type mtDNA are sufficient to modulate the effects of MELAS (Mitochondrial Encephalopathy, Lactic acidosis, and Stroke-like episodes).

## 6. Safety of mitochondrial transplantation

Enzyme-linked immunosorbent spot (ELISpot), enzyme-linked immunosorbent assay (ELISA), fluorescence-activated cell sorting (FACS) and multiplex analysis has demonstrated there is no direct or indirect immune response and there are no inflammatory effects associated with mitochondrial transplantation (13, 26, 28, 31).

Masuzawa et al. (13), showed using serial ELISA analysis that there was no significant increase in TNFα, IL-6 or high-sensitive c-reactive protein at 1-, 3- and 7- days post mitochondrial transplantation and provided evidence suggesting that the level of inflammation was ameliorated by mitochondrial transplantation. RNAseq analysis confirms this observation as a cardio-protective mechanism associated with mitochondrial transplantation in the heart (15).

To confirm the ELISA results Masuzawa et al. (13) also performed multiplex analysis of cytokines and chemokines. In this assay, both intact mitochondria and sonicated mitochondria were used with *in vitro* analysis. The sonicated mitochondria were used to determine the effects of mitochondria degradation products. Separate analysis was performed to determine innate chemokine and cytokine activation in human peripheral blood mononuclear cells. Masuzawa et al. (13) showed that there was no upregulation of cytokines associated with the immune response (IL-1, IL-4, IL-6, IL-12, IL-18, IP-10, macrophage inflammatory protein (MIP-1α and MIP-1β). Importantly, Masuzawa et al. (13) showed that mitochondrial transplantation upregulated epidermal growth factor (EGF), growth-related oncogene (GRO), IL-6 and monocyte chemotactic protein-3 (MCP-3). These cytokines have been shown to be associated with enhanced post-infarct cardiac function.

Ramirez-Barbieri et al. (31) investigated alloresponse and allorejection to syngeneic and allogeneic mitochondrial transplantation. This study examined immune response to single injections of mitochondria at concentrations of 1 × 10^5^, 1 × 10^6^ and 1 × 10^7^ and serial injections given a concentration of 1 × 10^7^ mitochondria on days −6, −3 and day 0. These concentrations are equivalent to 10-, 20- and 30-fold, and 90-fold respectively, the concentration of mitochondria used in our animal and clinical studies (13, 14, 26–28, 58, 74, 77, 92). Ramirez-Barbieri et al. (31) investigated both syngeneic and allogeneic mitochondria. Experiments were conducted using the BALB/cJ mouse strain to allow for human relevance. Allogeneic mitochondria were obtained from C57BL/6J mice. Mitochondria were delivered by intraperitoneal injection to maximize immune reaction. This was done as previous studies have shown that by intraperitoneal injection elicits a greater immune reaction than either intra-venous or direct injection by creating a greater distribution of the antigen to the lymph nodes and to different organs in the body. Immune response was measured at 10–17 days post-injection as the immune response in the BALB/cJ mouse strain is not evident prior to 7 days post antigen presentation.

Using this stringent protocol, Ramirez-Barbieri et al. (31) were able to show that there was no detectable direct or indirect B-cell or T-cell response as determined by ELISpot and FACS analysis and that multiplex analysis did not detect any increase in any of the cytokines or chemokines associated with the innate or acquired immune response for either syngeneic or allogeneic mitochondrial transplantation. Ramirez-Barbieri et al. (31) also showed there was no mitochondrial DAMPs (damage-associated molecular patterns) response associated with mitochondrial transplantation, no evidence of myocardial cellular damage or increased collagen content and no increase in circulating free mitochondrial DNA.

Masuzawa et al. (13) also showed there was no autoimmune response to mitochondrial transplantation and that there were no detectable anti-mitochondrial antibodies.

## 7. Biodistribution

Direct injection, intra-coronary, pulmonary and intra-renal artery infusion of mitochondria have been shown to provide discrete mitochondrial uptake. Once delivered the mitochondria rapidly enter the cells and remain present for at least 28 days, the terminal time for our animal experimentation. In a series of studies, separate analysis using autologous or xenogeneic human mitochondria each labelled with ^18^F-Rhodamine-6G (^18^F-R6G) have been performed. Results from these studies, in the *in vivo* swine model, have shown that the mitochondria are rapidly taken up by the cells in the end organ and are not present in other tissues. This localization of mitochondria following delivery was confirmed using mitochondria at concentrations 6-fold greater than we recommend. In our heart studies PET imaging demonstrated that intracoronary delivery distributes mitochondria specifically to the cardiac vascular supply, displaying signals only in the left ventricle when mitochondria were injected into the left coronary ostium. The tracer signal was not detected in other organs, despite the injection of much higher concentrations (six fold) of mitochondria than that used for therapeutic dosage. Similar findings were observed in the lung by pulmonary artery delivery (76), and in the kidney by intra-renal infusion (29), respectively.

Our studies show that in the heart, lung and kidney mitochondria rapidly crossed the vascular endothelial cells. ^18^F-Rhodamine-6G labeled mitochondria were found inside cardiomyocytes at 10 min following coronary artery delivery, while in the kidney the mitochondria were distributed throughout the tubular epithelium of cortex and medulla following renal artery delivery (28, 29). In the lung the transplanted mitochondria were detected within and around lung alveoli and connective tissue (76).

The mechanism(s) of vascular extravasation of mitochondria remain to be fully elucidated; however, the rapidity of mitochondria transport to cardiac cells is likely to involve mechanisms similar to bacterial or viral uptake.

## 8. Mechanisms of mitochondrial transplantation

We now have sufficient experimental proof to speculate on the overall mechanisms of mitochondrial transplantation.

### 8.1. Reactive oxygen Species

McCully et al. (14) investigated some of the mechanisms that may be involved in mitochondrial transplantation. These early experiments showed there was no increase in organ specific reactive oxygen species associated with mitochondrial transplantation. Thiobarbituric acid reactive substances (TBARS) assay, of lipid peroxidation showed that mitochondrial transplantation significantly decreased reactive oxygen species. Collaborative experiments using the reactive oxygen species scavenger, *N*-(2 mecaptopropionyl) glycine (MPG) when used throughout reperfusion or when added to mitochondria also failed to block the cardioprotection afforded by mitochondrial transplantation. Myocardial cell function as determined by sonomicrometry and cellular viability (necrosis and apoptosis) by triphenyl tetrazolium chloride staining was not altered with the addition of MPG.

These studies showed that mitochondrial transplantation did not increase reactive oxygen species (ROS) and that ROS was not involved in the mechanisms associated with mitochondrial transplantation. These initial findings agree with that of Kim et al. (63) who showed that mitochondrial delivery via centrifugal force did not cause intracellular damage, increase in oxidative stress (intracellular ROS and mROS) or apoptosis. Recently, Rossi et al. (25) have also shown that mitochondrial transplantation was associated with lower ROS production. The authors showed that mitochondrial transplantation decreased ROS generation as determined by the ROS-sensitive fluorescent probe MitoSOX together with the coherent decrease of TBARS production. In total these studies indicate that the effects associated with mitochondrial transplantation are not modified by reactive oxygen species. We have done no experiments using anti-oxidant enzymes.

### 8.2. Inflammation

It has been suggested that inflammation due to an acute immune response and inflammatory macrophage activation may play a role in tissue repair (93). Masuzawa et al. (13) has shown using serial blood samples over 4 weeks of recovery, that TNFα, IL-6 and high sensitivity C-reactive protein, sensitive markers of inflammation were significantly decreased in hearts receiving mitochondrial transplantation as compared hearts receiving vehicle alone suggesting that the level of inflammation was ameliorated by mitochondrial transplantation. Similar results were observed by Kaza et al. (26) who, using multiplex assay showed that there was no immune or inflammatory response or cytokine activation associated with mitochondrial transplantation.

Ramirez-Barbieri et al. (31) have shown by ELISpot assay that inflammatory cytokines INFγ, IL-2 (type 1 cytokines) were not increased with mitochondrial transplantation even at 90- fold the recommended mitochondrial concentration. Ramirez-Barbieri et al. (31) also examined cytokine profiles, involving a population of macrophages, Th1, Th2 cytokines. Multiplex analysis demonstrated that there was no detectable increase in the levels of any cytokine for either syngeneic or allogeneic mitochondria transplantation at any mitochondrial concentration (1 × 10^5^, 1 × 10^6^ or 1 × 10^7^). Guariento et al. (94) have shown in a clinical study of patients requiring extracellular membrane oxygen support for postcardiotomy ischemia-reperfusion injury, that mitochondrial transplantation was not associated with inflammatory response. In total, these studies indicate that the effects associated with mitochondrial transplantation are not modified by inflammation.

### 8.3. Adenosine receptors and K_ATP_ channels

An interesting phenomenon was observed with intracoronary delivery of mitochondria, namely a sustained increase in coronary blood flow. This effect on coronary blood flow was immediate and concentration-dependent, with maximal hyperemia achieved with an intracoronary injection of 1 × 10^9^ mitochondria (28). The increase in coronary blood flow was also accompanied by an increase in systolic shortening with no change in heart rate or mean arterial pressure. The mitochondria-induced increase in coronary blood flow was achievable only through intracoronary delivery of intact, respiration-competent mitochondria. Direct injection of mitochondria into the heart muscle or intracoronary delivery of devitalized mitochondria or intact HeLa p0-mitochondria, which lack respiration capacity, had no effect on coronary blood flow or systolic shortening. This finding is consistent with earlier findings by us and others that the transplanted mitochondria must be intact and respiratory competent.

**Figure 3:**
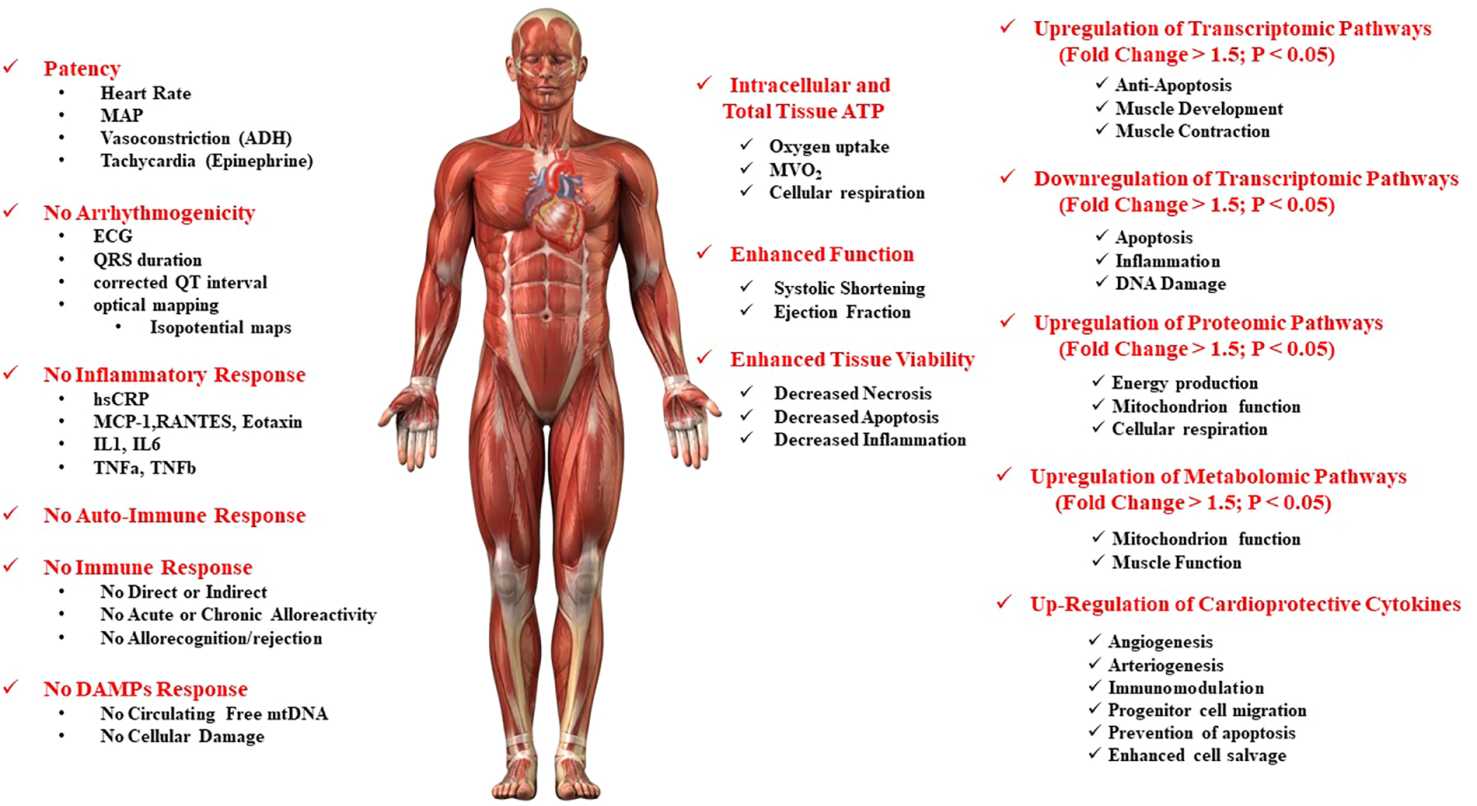
Safety and putative mechanisms associated with mitochondrial transplantation in the ischemia-reperfusion injured heart.

The mechanism for this was found not to be attributable to ATP (adenosine triphosphate) produced by the mitochondria or changes in oxygen saturation. In vivo inhibition of key coronary vasodilatory pathways: nitric oxide synthase (NOS), cyclo-oxygenase (COX), adenosine-receptors, potassium-ATP (K_ATP_) channels and oxygen saturation were also found to have no effect on the changes in coronary blood flow and systolic shortening. Interestingly, mitochondria-induced coronary vasodilation was attenuated in part by the inhibition of the inward-rectifying potassium (K_IR_) channels, consistent with studies that implicate K_IR_-channels in mechanisms of ATP-mediated vasodilation (28). These results are in agreement with earlier studies (14) that showed that in the Langendorff perfused heart with the non-selective adenosine receptor inhibitor 8-sulfophenyltheophylline or the non-selective potassium-ATP (K_ATP_) channel blocker glibenclamide or pre-incubation of isolated mitochondria with these drugs had no effect on the observable increases in systolic shortening and decreased infarct size obtained by mitochondrial transplantation (Figure 3). For review of the role of potassium-ATP (K_ATP_) channels the reader is directed to (8, 95).

### 8.4. Chemokines, cytokines

Masuzawa et al. (13) showed that there was up-regulation of cardioprotective cytokines. These cytokines, epidermal growth factor (EGF), GRO, IL-6 and monocyte chemoattractant protein-3 (MCP-3) have been shown to play key roles in angiogenesis, arteriogenesis, immunomodulation, progenitor cell migration, prevention of apoptosis and enhanced cell salvage and post-ischemic functional recovery. EGF has been shown to play a key role in ischemic injury protection in the heart by stimulating cell growth, proliferation, and migration. After cardiac infarction, GRO participates in the improvement in function and reconstitution of tissue mass and acts with IL-6 as a chemo-attractant which allows for enhanced vascularization, protection against cardiomyocyte apoptosis, and improved functional cardiac recovery. These chemokines have been shown to act with MCP-3 to enhance post-infarction cardiac function and improve cardiac remodeling independent of cardiac myocyte regeneration.

### 8.5. ATP

We and others have shown that mitochondrial transplantation improves bioenergetics and oxygen consumption both *in vitro* and *in vivo* (19–21, 23, 25). These beneficial effects are dependent upon the respiratory capacity and integrity of the transplanted mitochondria (13, 21, 28, 39).

We have demonstrated in the *in vivo* model that the mechanism of action of mitochondrial transplantation involves in part the prolonged increase in total tissue ATP content. Doulamis et al. (15) has shown that in the *in vivo* heart, mitochondrial uptake in the area at risk is evident at 2 h, 3 days and at 28 days post mitochondrial transplantation and is associated with significantly increased total tissue ATP content at both 2 h and at 28 days. The mechanism for this increase has been investigated by Rossi et al. (25) who have shown that mitochondria transplantation in a model of ischemia-reperfusion injury was able to restore the activity of the TCA cycle enzymes citrate synthase, alpha-ketoglutarate, succinate, and malate dehydrogenase and the enzymes of the electron transport chain, leading to increased intracellular ATP levels such that there was no difference as compared to non-ischemic controls. This agrees with the findings of Masuzawa et al (13), who showed that oxygen consumption rate in cardiomyocytes was significantly increased with mitochondrial transplantation and with Guariento et al. (58) who showed that mitochondrial transplantation significantly increased myocardial oxygen consumption.

### 8.6. Transcriptomic, proteomic and metabolomic responses to mitochondrial transplantation

Previously we have shown by microarray and proteomic analysis that cardioprotection following ischemia reperfusion injury is modulated by RNA- and protein-dependent mechanisms (12, 96). Transcriptomic and proteomic enrichment analyses indicated that ischemia downregulated genes/proteins associated with mitochondrial function and energy production, cofactor catabolism, and the generation of precursor metabolites of energy. In contrast, cardioprotection with cardioplegia significantly increased differentially expressed genes/proteins associated with the mitochondrion and mitochondrial function and significantly upregulated the biological processes of muscle contraction, involuntary muscle contraction, carboxylic acid and fatty acid catabolic processes, fatty acid b-oxidation, and fatty acid metabolic processes (12, 96). The transcriptomic and proteomic data demonstrated that the mitochondrion plays a significant role in both ischemia and in cardioprotection.

To ascertain the early expressed underlying global transcriptomic, proteomic and metabolomic changes conferred by mitochondrial transplantation we have performed RNAseq, SOMAscan and Metabolomic analysis to identify pathways up- and down-regulated with mitochondrial transplantation.

Masuzawa, et al. (13) showed that mitochondrial transplantation was associated with up-regulation of proteomic pathways. These *in situ* experiments demonstrated that mitochondrial transplantation beneficially altered proteomic pathways early in reperfusion, allowing for enhanced post-ischemic functional recovery and enhanced post-ischemic myocellular viability. Functional annotation clustering (*p* < 0.05, Enrichment Score > 2.0) indicated that the mitochondrion, the generation of precursor metabolites for energy and cellular respiration were enriched with mitochondrial transplantation and there were no down regulated clusters.

Doulamis et al. (15) also showed that mitochondrial transplantation up-regulated (fold change >1.5, *p* < 0.05) proteomic pathways for multicellular organismal processes, response to organic substance, stimulus and external stimulus, and multicellular organ and system development at 2 h and at 28 days recovery. All these pathways are associated with mitochondrial function and biosynthesis. Gene ontology localization analysis showed that the modulations in transcriptomics were affected by mitochondrion. No other organelle was implicated.

Doulamis et al. (15) showed that the changes in transcriptomics were consistent with proteomic alterations and showed that biological processes for regulation of multicellular organismal processes, regulation of biological quality, regulation of system processes, regulation of signaling, response to organic substance and response to oxygen containing compound were significantly upregulated in both RNA-seq and proteomic analysis.

These studies agree with the earlier studies by McCully et al. (96) and Black et al. (12) confirming the role of the mitochondrion in ischemia and in cardioprotection. These studies are also consistent with the findings of Rossi et al. (25) who have shown that mitochondrial transplantation up-regulates transcriptomic pathways associated with mitochondrial biogenesis [Peroxisome proliferator-activated receptors (PPAR) pathway], mitochondrial metabolism (IL-17, Ca2+, cAMP, and cAMP response element (CREB) signaling.

Cumulatively, these findings support the observations of Guariento et al. (58) who demonstrated that mitochondrial transplantation enriched metabolomic pathways for mitochondrion function and muscle function.

The physiological, functional, and biochemical results obtained in our animal studies in the ischemic-reperfused heart model support the pathways suggested by transcriptomic, proteomic and metabolic analysis. Our data clearly show that there is no immune or inflammatory response associated with mitochondrial transplantation. In agreement with the findings of Alexander et al. (81) who investigated mitochondrial transplantation by nasal delivery and showed that there was no evidence for activation of inflammatory pathways in the brain following mitochondrial transplantation and that there was no up-regulation of transcriptomic inflammatory signaling. The authors showed that the top canonical pathways upregulated by the nasal administration of mitochondria were the Nrf2-mediated oxidative stress response, along with telomerase, ERK/MAPK and synaptogenesis signaling. It was speculated that Nrf2-mediated response may regulate antioxidant proteins towards minimizing oxidative damage and that protein repair and clearance may also be triggered by ubiquitination, proteosome degradation and regulation of chaperone and stress response proteins. The authors suggested that mitochondrial transplantation may repair the acceptor cells like neurons, macrophages, and GFAP + cells possibly by changing their metabolic programming towards restoration of the damage and/or a more restorative phenotype.

The down-regulation of pathways for DNA damage are supported by the findings of Pacak et al. (20) who showed that mitochondrial transplantation rescued cell function and replaced mtDNA.

Down-regulation of pathways for proteolysis and apoptosis and up-regulation of the pathway for anti-apoptosis with mitochondrial transplantation also agree with our findings that mitochondrial transplantation significantly decreases myocardial necrosis and apoptosis (13–15, 26–28, 58, 77). Reduction in myocardial injury has been confirmed by significant decreases in CK-MB and cTnI and a significant decrease in caspase-3 like activity.

Up-regulation of transcriptomic pathways for muscle contraction and muscle development and proteomic pathways for muscle function and metabolomic pathways for muscle function also agree with our measured contractile indices where we have shown that mitochondrial transplantation enhances post-ischemic myocardial contractile function that includes increased left ventricular developed pressure, maintenance of left ventricular end diastolic pressure, increased systolic shortening, increased ejection fraction.

The up-regulation of proteomic and metabolomic pathways for energy production, mitochondrial function, cellular respiration and mitochondrial function and the metabolic pathway for mitochondrial function agree with our studies showing that mitochondrial transplantation increases total tissue ATP content, MVO2 and cellular respiration.

In total, these data show that the responses to mitochondrial transplantation are rapid and enduring. Changes in transcriptomics are evident at 2 h and remain upregulated for at least 28 days, the extent of our current experimental recovery duration in our animal studies. Transcriptomic and proteomic changes occur rapidly and persist for at least 28 days.

## 9. Conclusion

**Figure 4:**
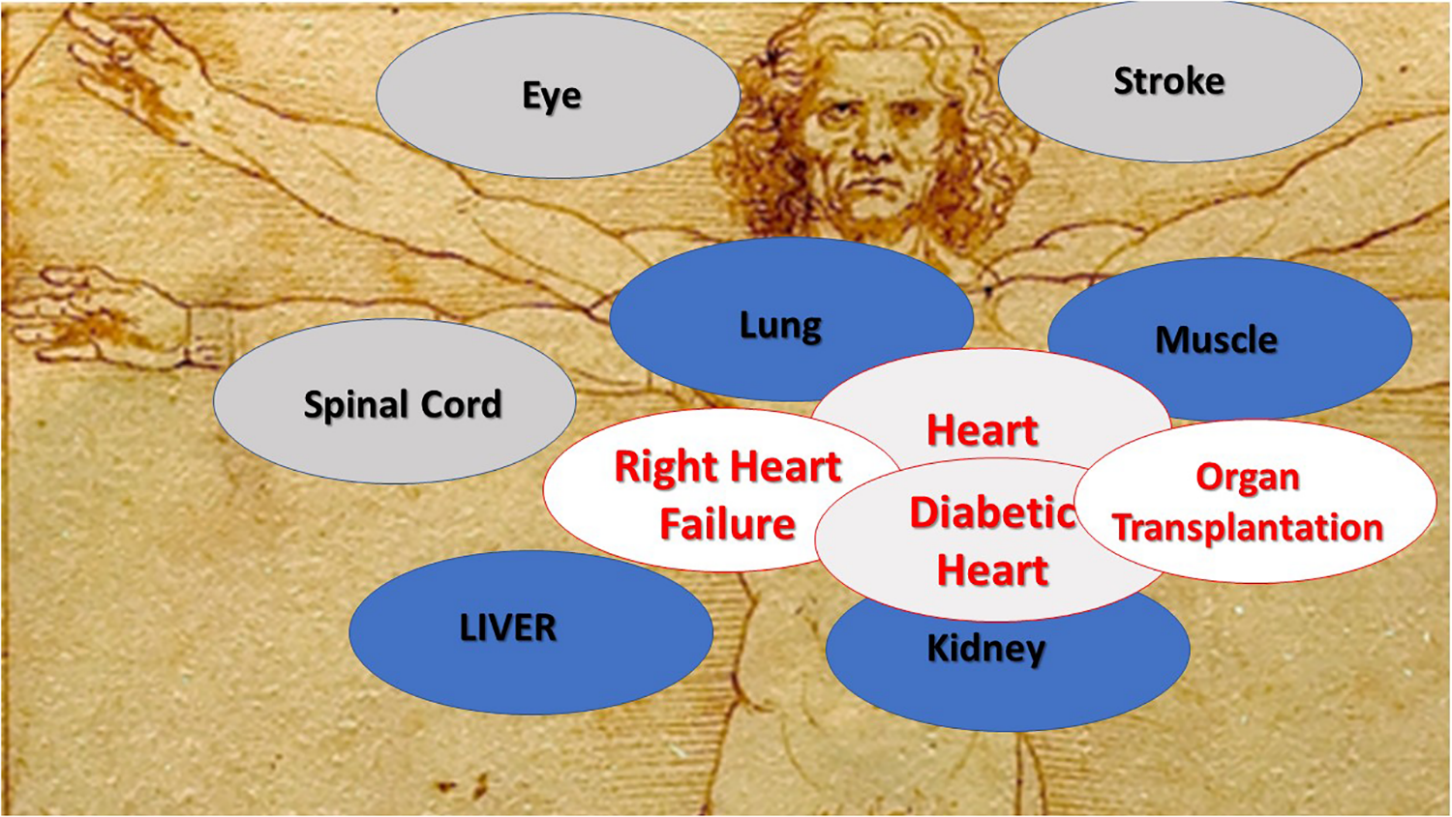
Clinical uses for mitochondrial transplantation.

The uses for mitochondrial transplantation are increasing. Mitochondrial transplantation has been used in the heart, lung, kidney, liver, skeletal muscle, brain and the eye (Figure 4). The importance of mitochondria as a therapeutic target and mitochondrial transplantation as a therapeutic modality is evident and is expanding to involve a myriad of pathologies. Specific usage and procedural indices have been suggested and further modification and elucidation will occur. The data to date suggests that mitochondrial transplantation may provide new and improved approaches to many pathologies and conditions. This review provides the observations obtained from our studies with those of others in the area and does not include the many variations now being proposed. We hope that mitochondrial transplantation as a methodology will continue to increase in usage and for disease and non-disease states and that this review will stimulate further investigation.
